# Harnessing the Four Elements for Mental Health

**DOI:** 10.3389/fpsyt.2019.00256

**Published:** 2019-04-24

**Authors:** Jerome Sarris, Michael de Manincor, Fiona Hargraves, Jack Tsonis

**Affiliations:** ^1^NICM Health Research Institute, Western Sydney University, Westmead, NSW, Australia; ^2^Professorial Unit, The Melbourne Clinic, Department of Psychiatry, Melbourne University, Melbourne, VIC, Australia; ^3^THRI, Western Sydney University, Campbelltown, NSW, Australia

**Keywords:** lifestyle, mental health, mood, anxiety, psychological, well-being, nature, lifestyle medicine

## Abstract

Humans are intimately connected to nature, and our physical and mental health is influenced strongly by our environment. The “elements,” classically described in humoral theory as Fire, Water, Earth, and Air, all may impact our mental health. In a contemporary sense, these elements reflect a range of modifiable factors: UV light or heat therapy (Fire); sauna, hydrotherapy, and balneotherapy (Water); nature-based exposure therapy and horticulture (Earth); oxygen-rich/clean air exposure; and breathing techniques (Air). This theoretical scoping review paper details the emerging evidence for a range of these elements, covering epidemiological and interventional data, and provides information on how we can engage in “biophilic” activities to harness their potential benefits. Interventional examples with emerging evidentiary support include “forest-bathing,” heat therapy, sauna, light therapy, “greenspace” and “bluespace” exercise, horticulture, clay art therapy activities, and pranayamic yoga breathing exercises. Further robust research is however required to firmly validate many of these interventions, and to establish their therapeutic applications for the benefit of specific mental health disorders.

## Introduction

Humans have an intimate connection to nature, and by our very being, we are part of nature (1). Several distinguished clinicians and historians in centuries past have posited that primordial elements construct a person and that imbalances are the cause of ill health. While scientific advancement has moved well beyond such rudimentary medical theory, there may still be merit in considering some of the basic tenets of the philosophy underpinning the humors for potential application in maintaining or enhancing mental health. At the very least, it should be recognized that some aspects of modernity have had a deleterious effect on mental health (2, 3). While a range of medications have been invented, these have had only modest effects for most psychiatric disorders, and concerns have increased over the impact of negative psychosocial changes such as the breakdown of family units, stressful jobs and a challenging work–life balance, a more sedentary life, poorer nutrition, declining air quality, and a decreased connection with nature. In respect to the diminishing interface with our biosphere than was evident with our ancestors, this is also affecting our microbiome, which is modified by exposure to nature (4).

The classical understanding of the elements and their relationship with human health was advanced by physicians such as Hippocrates and Galen (5). This was characterized as the “four humors,” which each pertained to an element with distinct qualities: Melancholic [Earth (dry)], Sanguine [Air (cold)], Choleric [Fire (hot)], and Phlegmatic [Water (moist)]. An imbalance of these elements internally was considered to be responsible for disease. There was also appreciation that external exposure to these elements could modify health *via* redressing imbalance. For example, if a person had signs of melancholia (dry skin, feeling cold, emotionally withdrawn, reduced mobility, and depressed mood), then exposure to warmth, moisture, and activity may be considered to be of assistance. Traditional medicine models including Unani, Ayurveda, and Traditional Chinese Medicine also embodied similar elemental constructs and are still practiced in modern times, with health conditions treated by addressing elemental imbalances and deficiencies (6).

Firstly, *we are not proposing that therapeutic use of “the elements” should replace mainstream pharmacotherapy or psychological techniques*. We are, however, suggesting that enhanced contemporary understanding (and requisite robust research) of this interplay may inform a potential use within an integrative treatment model for mental disorders. This may provide an additional avenue to enhance general mental health and perceived well-being. In the most basic form, as humans, to survive (and indeed thrive) we need clean air, fresh water, a regulated body temperature, and the nutrients that are ultimately derived from the earth. As our understanding of the importance of these aspects on mental health is advancing, so is the consideration of harnessing their benefits for use as potential health-enhancing interventions.

**Figure 1:**
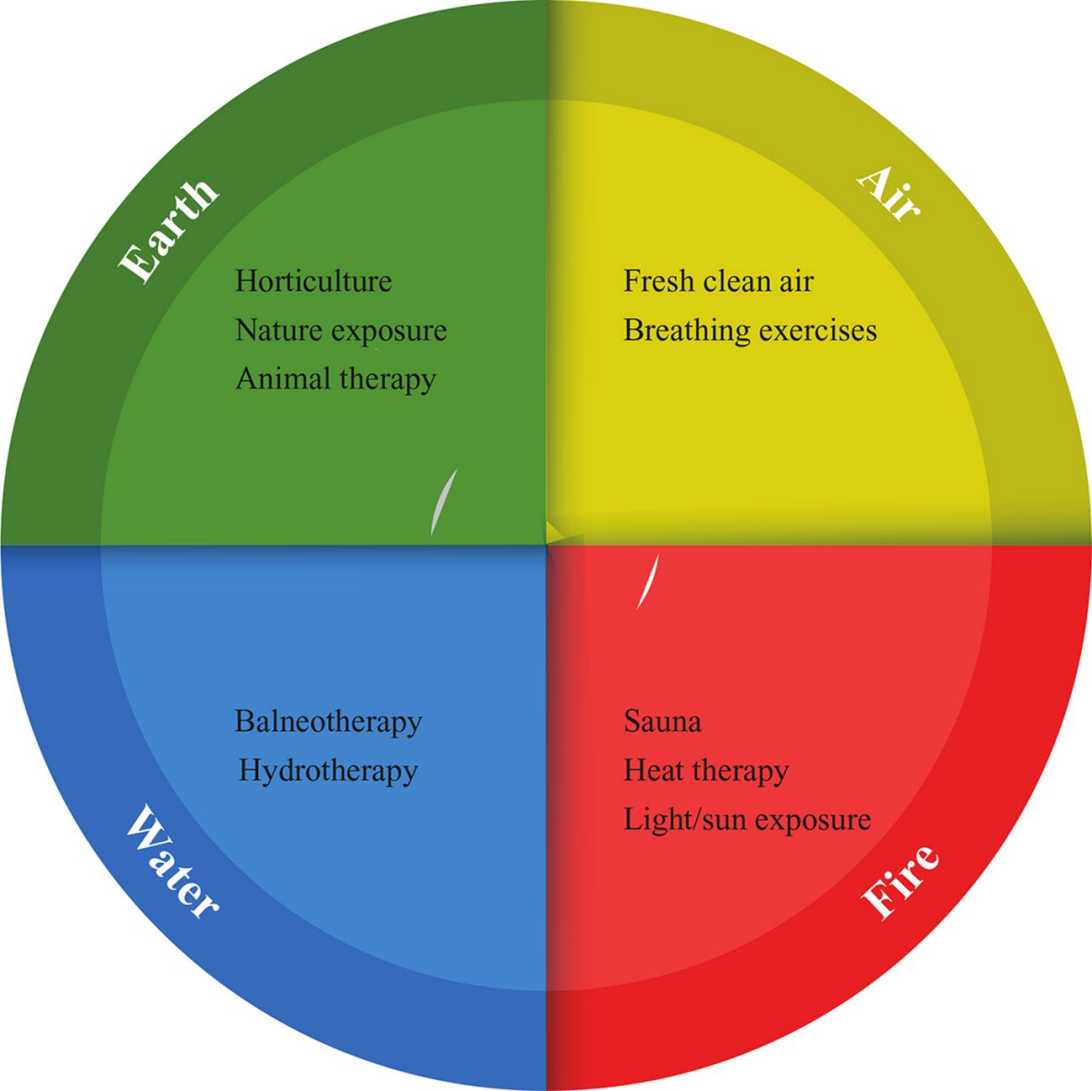
Four elements (humors) and mental health applications.

To our knowledge, no academic paper to date has covered a review of the evidence for the mental health applications of all “the elements” in this classic sense; thus, we conducted a broad scoping review of the area. We reviewed pertinent literature from Medline, EBSCO, and Web of Science databases, and selected key literature that best fitted thematically within the domains of “Earth,” “Fire,” “Water,” and “Air.” **Figure 1** details the therapeutic interventions covered under these domains. We specifically cover the key literature on 1) the relationship between these elements and mental health (in respect to underlying epidemiological data) and 2) any clinical trials utilizing interventions that fitted thematically under one of the four elements.

## Earth

We categorized this domain to encapsulate aspects pertaining to the influence of direct exposure to earth/soil and flora, time spent in nature (in particular wilderness environments) and the application of “greenspace exercise,” interactions with animals, and novel interventions such as clay art therapy.

Adequate exposure to nature (greenspace) may provide benefits for general health, and data support that increased urbanization and exposure to excessive industrialization may negatively impact health (7). The benefits of spending time in nature for mental health are evident (8), including increased exposure to sunlight and fresh air, in addition to a range of beneficial psychosocial elements in some situations. Aside from these benefits, direct interaction with nature (and biodiversity) also impacts the development of the microbiome, which may also have mental health influences (9, 10). While this may be beneficial, when interacting with soil, some caution is needed in terms of potential contaminants (e.g., chemicals and harmful pathogens).

In respect to interventions within this domain, a novel study involving “clay art” therapy conducted a short-term intervention (six 2.5-h sessions) assessing its effects on a range of mental health outcomes for 106 depressed individuals (11). Compared to the visual art group control, depressive symptoms decreased, in addition to improvements in general health and perceived well-being. The therapeutic effects of sufficient greenspace may involve a range of factors, and it is of note that, in children, it also stimulates cognitive development *via* increased scope for risk-taking, imagination, and self-discovery (12).

Actively engaging in horticulture may also improve mental health. A nature-assisted rehabilitation program in a group of general patients (*n* = 118) with noted severe stress levels and/or mild to moderate depression was assessed for changes in sick-leave status and healthcare consumption (13). These patients were compared to matched controls (*n* = 678). Results showed that there were benefits in reduced healthcare consumption for those participating in the rehabilitation garden compared to the control, but no change in sick-leave status.

In respect to being a potential adjunctive intervention in people with a range of psychiatric disorders, a Serbian study was conducted involving 30 patients who participated in a horticultural therapy (HT) program or a control group (occupational art therapy) (14). The results indicated that HT had a positive influence on the mental health and well-being of the participants. Furthermore, a difference was revealed in the test results of the stress subscale before and after the intervention, with a stronger effect for anxious males. Another HT project involving 49 participants undergoing a Veterans Affairs substance abuse treatment program showed that 5 h/day of HT for 3 weeks had a reduction in cortisol levels and a trend for improvement in depressed mood and quality of life compared to usual occupational therapy (15). Other uncontrolled research involving a 12-week HT program (*n* = 46) for people with clinical depression confirmed significant beneficial change in mental health variables during the intervention (16). A reduction in depression severity persisted at 3-months follow-up, and most interesting, an increase in social activity and social cohesion was reported after the intervention. An 8-week indoor gardening program has also revealed beneficial social connectedness and a reduction in loneliness in the elderly confined to nursing homes (17). Further research has shown that compared to a sedentary activity, such as reading, it can even have acute stress-reducing effects, as seen in salivary cortisol reductions (18).

To increase a mental health benefit from exposure to nature, physical activity may also be coupled with this (e.g., nature walks). Such exercise may synergistically increase well-being beyond physical activity in an urban setting (19). While the evidence of specific health benefits from nature-assisted therapy (e.g., wilderness treks) is unclear due to a current lack of rigorous studies, overall the evidence is supportive of a therapeutic effect. A meta-analytic analysis of 10 UK studies involving a clinical population of participants (*n* = 1,252) with a range of mental disorders found moderate effect sizes for green exercise in improving mood and self-esteem in healthy samples (19). This result also mirrored a systematic review by Annerstedt and Wahrborg (20) who found that for studies of moderate to low evidence grade, health improvements were reported in 26 out of 29 studies. Finally, a systematic review of 11 studies (*n* = 833) on participation in physical activity and exercise in natural environments (versus indoor activity) revealed that 9 out of 11 trials showed some improvement in mental well-being (21). Compared to exercising indoors, exercising in natural environments was associated with decreases in tension, confusion, anger, and depression, and a perceived increase in energy and feelings of positive engagement. Specific examples of nature-based physical activity, with a direct connection to soil or flora, that have shown to enhance mental health outcomes include sailing, gardening, horse-riding, wilderness hiking, and running in nature (19). Green exercise has also been found to be an effective way to reduce the stress of workers, showing an improvement on positive affect and blood pressure beyond urban indoor exercise, in a small-scale acute pilot study (*n* = 14) (22).

One interesting public health application found to occur in Japan (23) is the prescriptive recommendation of “forest bathing,” known as “*Shinrin-yoku*.” In simple terms, this involves advising people to spend time in forests. Preliminary evidence reveals that such exposure (compared to time spent in urban settings) can improve general mental health while altering physiological stress and immune biomarkers. Studies have, for example, revealed that short-term exposure to forests reduces levels of malondialdehyde, interleukin-6, tumor necrosis factor alpha, and cortisol, and enhances mental health indices (24). The same research group found a similar beneficial effect in elderly people with chronic heart failure (25). Forest-based therapy has also been found to be beneficial for treating depression and anxiety symptoms in patients with chronic stroke (26). One study involving 55 patients with chronic stroke were randomly assigned to either a stay at a recreational forest site or at an urban hotel. The results revealed lower depression, anxiety, and stress scores in favor of the forest stay group.

Humans commonly have close relationships with animals, and they can provide physical affection and a feeling of unconditional love, assist in the maintenance of a routine, and also provide responsibility and an additional sense of life purpose. Formalized animal-assisted therapy may involve horses (equine therapy), dogs, or even interactions with mammals such as dolphins. Time spent with farm animals by people with a range of psychiatric disorders may reduce depression and state anxiety and increase a sense of self-efficacy (27).

## Fire

We categorized this domain to encapsulate aspects pertaining to the influence of direct exposure to light (sunlight or artificial light) or heat (*via* vectors including sauna, or heated objects applied to the body, e.g., hot rocks).

It is a commonly held transcultural anecdotal belief that one’s mood improves when the sun is out, and inclement weather is at bay. While acute exposure to sunshine (and a pleasant temperature according to the individual) may enhance mood, there is an inconsistent relationship with seasonal variations with the prevalence of mood disorders (28, 29). The effects of sunshine exposure on mental health may in part be mediated by vitamin D. However, while low levels of vitamin D appear to be associated with depression risk, there is conflicting evidence as to the effects of supplementation on improving mood (30–32). In fact, one study involving 198 participants with multiple sclerosis who were followed prospectively for an average of 2.3 years revealed that reported sun exposure was inversely associated with depression and fatigue scores (33). Interestingly, only high levels of vitamin D (> 80 nm) were inversely associated with depression scores, but this was not significant after adjustment for reported sun exposure. In other words, the sun exposure, not the vitamin D levels, reflected improvement in mood and energy.

Another facet of “light-based therapy” for mood modulation is in the application of simulating dawn in order to stimulate serotonin and cortisol secretion and to regulate circadian rhythm (34, 35). Several clinical trials have shown that this may be particularly beneficial for seasonal affective disorder (SAD). Randomised controlled trials (RCTs) of 2 to 8 weeks in duration have assessed the differential effectiveness between simulating dawn (half an hour prior to waking between 100 and 300 lux) versus high 1,500–10,000 lux bright light therapy (36–38). Results are conflicting, and both may be potentially effective in SAD, with stronger support for general bright light therapy.

One of the most common health interventions involving the targeted use of heat is sauna, which includes the traditional variety (using radiant heat and hot rocks to create moisture) and the modern infrared variety (which typically uses far-infrared light). To date, significantly more research exists with regard to traditional sauna (39), although emerging evidence suggests that infrared sauna promotes similar physiological benefits (40). While evidence remains preliminary with only a very small number of controlled studies having been conducted on the health effects of regular sauna bathing (40), the basic physiological effects have been known for some time (41).

More recently, the first longitudinal studies have been conducted on a large Finnish cohort (*n* = 2315), with results that show positive outcomes in a range of health domains, such a cardiovascular disease (42), hypertension (43), respiratory illness (44), and even dementia (45). The authors of these studies outline how the physiological response to sauna bathing corresponds to low- and moderate-intensity physical exercise and suggest that “these proposed functional improvements associated with sauna bathing correspond to similar benefits seen with regular physical exercise” (p. 546) (42). Although a current breadth of research is lacking (especially for mental health applications), because the general benefits appear to be associated with increasing activity of the thermoregulatory system, it is reasonable to assume that similar benefits are obtained from other modalities of atmosthermic bathing from different cultural traditions. These include the Islamic “hammam,” the Native American “sweat lodge,” and the Japanese “onsen,” all of which enjoy long-held reputations for their regenerative capacity (46).

One particular aspect of classic sauna practice that requires further interrogation for general and mental health effects is the common adoption of cold exposure after heat exposure (cold showers/air/snow after the sauna). A 2015 study involving 3,018 participants (aged 18–65 years) without severe comorbidity and no routine experience of cold showering were randomized to hot and cold shower cycles (or a control group) during 30 consecutive days followed by 60 days of showering cold at their own discretion for the intervention groups (47). The primary outcome was illness days and related sickness absence from work. Results revealed a 29% reduction in sickness absence for hot to cold showers compared to the control group, but not illness days in adults without severe comorbidity. These results suggest that further investigation into cold exposure and sauna bathing is an important area of research, especially considering the prevalence of the practice at a global level.

At least one small study exists (*n* = 9), but this examined acute body temperatures rather than longer-term effects and did not investigate qualitative differences in perception of sauna enjoyment as a result of cold exposure (48). Another study from 2016 (*n* = 37) found that cold water immersion after sauna may be safe for patients with chronic heart failure, but that the practice should be conducted with caution (49). The study likewise did not consider qualitative aspects of cold exposure, which has been described as eliciting perceived regeneration in recent ethnographic literature (50). While outside the focus of this review (and in direct contrast to heat based therapies), there is also a recognized potential use of cold therapy alone (cryotherapy) for a range of health applications.

In respect to specific effects on mental health outcomes from sauna, there is overwhelming anecdotal evidence from traditional folklore (46), but minimal scientific or sociological evidence. In the same Finnish cohort discussed above, there was found to be a strong association between frequent sauna bathing and a reduction of psychotic disorders after a median follow-up of 25 years (51), but this is the only longitudinal evidence currently available. Other studies have suggested a positive effect of mood from a Korean “jjimjilbang” sauna (52, 53), as well as reduction of pain intensity in chronic conditions such as hypertension headaches (54). Given the prevalence of anecdotal reports about the relationship between sauna and improved mental health, this is another promising line of investigation that requires sustained attention from the global scientific community, especially in light of recent positive results concerning the use of whole body hyperthermic (WBH) therapy to alleviate major depressive disorder (55). WBH involves the use of sustained heat for 1–2 h to raise core body temperature (sometimes *via* a device that heats the inside of a tent that covers the body).

A prior uncontrolled study found that a single session of WBH reduced depressive symptoms in people experiencing depression, and thus researchers sought to test whether this effect would outperform a sham control condition (a matched procedure but with no heat). They assessed a single acute treatment in a 6-week, randomized, double-blind study involving 34 adults with major depressive disorder (MDD) (55). Results revealed that compared with the sham group (mimicking all attributes accept the intense heat), just one WBH therapy treatment showed significantly reduced depression scores maintained across the 6-week post-intervention study period.

## Water

We categorized this domain to encapsulate aspects pertaining to the influence of direct consumption of water, and the exposure to water, including the use of general hydrotherapy, balneotherapy (treatment of disease *via* bathing in natural mineral springs), and water-based physical activity (56).

Mammalian life requires water for existence, with the human body being composed of approximately 60% water (57). Aside from the importance of adequate hydration for the proper electrolyte balance, intracellular function, and extracellular communication, there may be mood and cognitive consequences of dehydration (58). In respect to mental health effects, a novel study assessing mild dehydration (produced by intermittent moderate exercise) was conducted on 25 healthy females. Participants undertook three 8-h, placebo-controlled experiments involving different hydration states (based on exercise or diuretic-induced states) (59). While most aspects of cognition were not affected, significant adverse effects of 1.36% dehydration showed a degradation in mood, increased perception of task difficulty, lower concentration, and more headache symptoms. This equates to a moderate level of dehydration (many studies will not push the body past 1.50% dehydration due to an increasing range of negative consequences).

The therapeutic application of water is evident in many cultures, especially in respect to natural mineral spa bathing (commonly in hot springs). One study (*n* = 237) comparing balneotherapy in spa resorts with paroxetine in treating generalized anxiety disorder showed a significant advantage of the spa treatment as assessed on anxiety scales, with remission and sustained response rates also significantly higher in those treated with balneotherapy (60). When compared to progressive muscle relaxation for stress-relief, short-term balneotherapy provided higher subjective ratings of relaxation with healthy participants (*n* = 49) and was similarly beneficial in decreasing salivary cortisol (61).

Balneotherapy has also been shown to improve quality of life and symptoms of chronic pain in fibromyalgia patients. A study conducted at the Dead Sea for 10 days (*n* = 48) demonstrated significant improvements on all well-being measures (62). Further evidence from Ozkurt et al. (63) supported these positive effects for fibromyalgia sufferers (*n* = 50), with their 2-week treatment producing significant improvements on all outcome measures, including pain, fibromyalgia impact, depression, and quality of life. In both studies, follow-up assessments showed physical improvements lasting 3 months on average, while psychological improvements were shorter-lasting, suggesting the benefits of regular balneotherapy in managing fibromyalgia. The classical use of contemporary steam rooms also provides an ambient therapeutic interface with both heat and water.

While in-depth exploration of water-based physical activities are outside the auspices of this review, a couple of novel applications of immersive water-based activities are worth noting. A recent study evaluated Deptherapy, a UK-based charity that provides a scuba diving intervention as support to military veterans who experienced life-changing injuries from combat (64). A total of 15 male veterans were assessed in an uncontrolled format both prospectively and retrospectively on a range of quantitative measures of mental well-being and functional ability outcomes. Participants reported enhancement on a range of psychosocial well-being measures. The researchers posit that scuba diving has the potential in part to benefit injured veterans due to the requirement of complete focus and the feeling of weightlessness when underwater. Another water-based application is in the use of “surf therapy,” which has shown to improve participants’ well-being (65, 66). It is however recognized that this offers a range of other ancillary benefits (beyond direct effects from the immersion in water), including increased mindfulness and atelic skill development, exposure to fresh air and sunlight, and general physical activity.

## Air

We categorized this domain to encapsulate aspects pertaining to the influence of direct exposure to fresh clean air, conscious and more effective breathing, and specific breathing exercises (such as utilized in yoga, known as *pranayama*). This may be achieved *via* regulated breathing-focused biofeedback techniques (67), which may reduce anxiety and perceived stress. For instance, regulation of natural breathing synchronizes electrical activity in human piriform (olfactory) cortex, as well as modulating limbic-related brain areas (which includes amygdala and hippocampus; key brain areas affecting anxiety/stress, and memory) (68).

One key environmental factor that is recognized to influence physical health involves the increased exposure to particulate air pollution, which is one common issue of modernity (69). This has been found to have a potential profound effect on the central nervous system. Exposure to air pollution, for example, has been found in a longitudinal study of 537 elderly Koreans to be associated with an increase in depressive symptoms (70). Additionally, evidence from a cross-sectional study indicates that secondhand cigarette smoke is positively associated with increased depressive symptoms in smoking-naive people (even after adjustment for a range of demographic factors and comorbidities) (71, 72).

Emerging preclinical evidence suggests that air pollution may induce oxidative stress, neuroinflammation, microglial activation, and cerebrovascular dysfunction, while potentially altering the blood–brain barrier (73). For example, a mouse model study investigated whether long-term (10 months) exposure to ambient fine airborne particulate matter compared to filtered air affected depression-related animal behavior and cognitive responses (74). The data revealed that mice exposed to long-term air pollution displayed more depressive-like responses and impairments in spatial learning and memory as compared with mice exposed to filtered air.

As well as the quality of the air that we breathe, the way that we breathe is also associated with our health and well-being. While functional and dysfunctional breathing are difficult to define, dysfunctional breathing, including restricted, shallow, rapid, or irregular breathing, is implicated in a range of physical and psychological health conditions (67). In particular, dysfunctional breathing is recognized as symptomatic of depression and anxiety, and there is a corresponding high prevalence of depression and anxiety among people with chronic breathing disorders (75).

Mindful breath awareness (MBA) and breath regulation techniques (BRTs) have been reported as the most commonly used mind–body therapy by adults with medical conditions in the United States (76) and the second most commonly used of all complementary health approaches (second only after all natural dietary supplements combined) (77). MBA and BRTs are commonly used as components of psychological and complementary treatments for mental health conditions, including cognitive behavior therapy (CBT), mindfulness-based cognitive therapy (MBCT), mindfulness-based stress reduction (MBSR), and mind–body practices in general, including yoga, taichi, qigong, relaxation training, and mindfulness and other forms of meditation (78). Each of these has been studied in clinical research and included in numerous reviews (78–82).

Clinical studies that have focused on BRTs as a primary intervention for general mental health are mostly yoga-based breathing exercises. MBA and BRTs are integral to several aspects of yoga practice. For example, yoga postures are ideally done with MBA and coordination of breath with movements; specific yoga BRTs (known as *pranayama*), relaxation, and meditation techniques often use the breath as a means to relax and focus the mind. Several clinical studies have found that yoga BRTs were effective in reducing severity of depressive symptoms (83–87). One RCT using a yoga BRT technique, known as Sudarshan Kriya Yoga (SKY), found that the technique was equally as effective as both electroconvulsive therapy (ECT) and a commonly prescribed pharmacological antidepressant (imipramine) for clinical depression over a 4-week period (85). Later studies using the same yoga BRT also found reductions in depressive symptoms among alcohol-dependent participants following a detox program (87) and reductions in posttraumatic stress (PTS) and depressive symptoms among survivors of the 2004 Southeast Asia Tsunami (84). More recent studies that incorporate MBA into multi-component yoga interventions have also found benefits for reducing symptoms of depression and anxiety and improving well-being (83, 86). Streeter and colleagues have also found preliminary evidence that suggests that breath-centered yoga practices increase gamma amino-butyric acid (GABA) levels in the brain, which is associated with improvements in depression (88).

Several researchers have postulated neuro-physiological models to explain the benefits of breath-centered yoga practices in diverse, frequently comorbid medical conditions, including mental health disorders, based on the concept and evidence that yoga practices reduce allostatic load in stress response systems and restore homeostasis and balance in the human system (88, 89). They hypothesize that breath-centered yoga-based practices 1) correct underactivity of the parasympathetic nervous system (PNS), in part through stimulation of the vagus nerves, the main peripheral pathway of the PNS; 2) increase low levels of GABA; and 3) reduce the allostatic load of stress. Depression, anxiety, PTS, and chronic pain exemplify medical and mental health conditions that are exacerbated by stress, have low heart rate variability (HRV) and impaired GABAergic activity, respond to pharmacologic agents that increase activity of the GABA system, and show symptom improvement in response to breath-centered yoga-based interventions (88, 89).

## Discussion

As detailed above, the “elements” in both a classical and a contemporary sense have effects on our mental health and are potentially modifiable aspects that can be harnessed as therapeutic interventions. The most robust interventional evidence currently available shows tentative support for several use of the elements *via* horticultural and nature-exposure therapy, green exercise/physical activity, sauna and heat therapy, balneotherapy, and breathing exercises. It should be noted that, in many cases, these interventions were not studied in definitive diagnosed psychiatric disorders and thus it is premature to consider these therapies to be gold standard treatments. Regardless, the evidence does reveal many positive general mental health benefits. The mechanisms of action underpinning these lifestyle medicine approaches are varied (due to the breadth of interventions covered). More attention is required to tease out the direct health effect of the elements, as many interventions will have other confounding aspects such as physical activity, mindfulness, or attention-based activity, they may also have a social interface component, and finally commonly will have a range of nature-based environmental influences (involving increased exposure to sunlight, fresh air, etc). As detailed above, known health effects for example include early morning light increasing serotonin and cortisol production, modulation of core temperature boosting circulation and the immune response, breathing regulation affecting a range of neurochemical effects and HRV and blood flow dynamics, and exposure to flora and fauna altering the microbiome.

While it is understood that this paper does not follow a strict reductive analysis of specific techniques for specified psychiatric disorders, it is intended to provide a paradigm to be able to expand a different way of thinking, particularly for the consideration of novel interventions that may work alongside conventional treatments to enhance mental health. It is clearly accepted that many of the interventions do not currently possess firm evidentiary support, and further research employing randomized and controlled designs (ideally within mixed-methods designs) is required. Promising areas of future inquiry concern further research into the impact of the elements on mental health, in particular interventions such as sauna and heat therapy, hydrotherapy, and nature-exposure therapies.

There are many potential benefits of “harnessing the four elements” for health purposes. This approach can be recognized as being considered part of the emerging field of “lifestyle medicine.” Aside from providing the fundamentals of sustaining life, and the potential mental health benefits detailed above, there are a range of ancillary advantages. Such approaches may enhance social connectivity, improve physical health, may enhance brain health and cognition, and increase “self-efficacy” or mastery (90). These approaches are generally very low-cost, although choices may be limited in developing countries and those with overindustrialized, polluted environments. In respect to clinical considerations for more mainstream lifestyle medicine implementation, recent data from Sweden suggest that there are a range of barriers still persisting (91). Major factors for why lifestyle modifications are not being more commonly implemented in clinical practice include a lack of knowledge and roles, lack of organizational support and resources, a low perceived importance of these measures, and a deficit of time to provide the needed attention. Overcoming these barriers within the mental health sphere also presents with more challenges in respect to increased comorbidity, potential motivational issues, lower socioeconomic status in those with more severe mental illness, and a deficit in most jurisdictions of qualified clinical staff with lifestyle medicine-focused skills and training. While there is no easy solution at present, the increased academic emphasis on the emerging lifestyle medicine field should increase awareness and, with an evolving evidentiary base, be able to interface more profoundly with policy makers and clinical bodies.

One further “element” that may be considered within the elemental paradigm is the philosophical consideration of a fifth element, which has been termed as “void,” “space,” or “ether.” This element is generally not present in the Hippocratic–Western framework, yet it is found in other traditional systems such as Ayurveda. A more abstract-related connection to this concept is that of our understanding of space and the quantum physics field. Quantum theorists postulate the importance of quantum physics for cognitive neuroscience and psychiatry, and suggest that the laws of quantum mechanics may have an influence on the dynamics of consciousness and the nature of mind (92). A further curious factor relating to space and mental health is a well-documented phenomenon known as the “overview effect,” experienced by astronauts travelling into space (93, 94). Viewing the Earth from space has often prompted astronauts to report overwhelming emotion and feelings of identification with humankind and the planet as a whole. Psychological constructs, such as awe and self-transcendence, appear to contribute to a psychological understanding of the experience of vast open spaces. While such experiences may be associated with well-being and increased altruism and other prosocial behavior, in some cases they can also be associated with increased fear and anxiety (94). There are many factors associated with mental health, open spaces, and space travel, and they warrant further investigation for mental health implications, including for the majority of us who do not leave the planet.

At the heart of this paper is encouragement for the psychiatric field (and the broader medical field) to consider going back to basics in terms of understanding the importance and the potential utilization of the elements for mental health. Of course, we are not diminishing the importance of pharmacotherapy and psychological techniques for psychiatric disorders. We are however saying that nature still holds a key to addressing many of the woes that currently plague society. We are becoming increasingly more socially isolated and hyperstimulated, and connecting more intimately to the biosphere and our fellow humans will always provide mental health sustenance.

## Author Contributions

JS: lead author and contributor to the earth section. MdM: contribution to section on air. FH: contribution to section on water, referencing, and editing. JT: contribution to section on fire.

## Conflict of Interest Statement

JS has received presentation honoraria, travel support, clinical trial grants, book royalties, or independent consultancy payments from Integria Healthcare & MediHerb, SPRIM, Pfizer, Scius Health, Key Pharmaceuticals, Taki Mai, FIT-Bioceuticals, Blackmores, Grunbiotics, Soho-Flordis, Healthworld, HealthEd, HealthMasters, Elsevier, Chaminade University, International Society for Affective Disorders, Complementary Medicines Australia, Terry White Chemists, ANS, Society for Medicinal Plant and Natural Product Research, Sanofi-Aventis, Omega-3 Centre, the National Health and Medical Research Council, and CR Roper Fellowship.

The remaining authors declare that the research was conducted in the absence of any commercial or financial relationships that could be construed as a potential conflict of interest.
